# Combining Fluorinated Polymers with Ag Nanoparticles as a Route to Enhance Optical Properties of Composite Materials

**DOI:** 10.3390/polym12081640

**Published:** 2020-07-23

**Authors:** Veronica Satulu, Bogdana Mitu, Valentin Ion, Valentina Marascu, Elena Matei, Cristian Stancu, Gheorghe Dinescu

**Affiliations:** 1 National Institute for Lasers, Plasma and Radiation Physics, PO Box M G-36, 077125 Magurele, Romania; veronica.satulu@infim.ro (V.S.); valentin.ion@inflpr.ro (V.I.); valentina.marascu@inflpr.ro (V.M.); cristian.stancu@infim.ro (C.S.); dinescug@infim.ro (G.D.); 2 National Institute of Materials Physics, PO Box MG-7, 077125 Magurele, Romania; elena.matei@infim.ro

**Keywords:** magnetron sputtering, PTFE matrix, Ag nanoparticles, AgC_x_F_y_O_z_ bond, barrier layer, optical properties, surface plasmon resonance

## Abstract

Polymer-based nanocomposites have recently received considerable attention due to their unique properties, which makes them feasible for applications in optics, sensors, energy, life sciences, etc. The present work focuses on the synthesis of nanocomposites consisting of a polytetrafluorethylene-like matrix in which metallic nano-silver are embedded, by using multiple magnetron plasmas. By successively exposing the substrate to separate RF magnetrons using as combination of target materials polytetrafluorethylene (PTFE) and silver, individual control of each deposition process is insured, allowing obtaining of structures in which silver nanoparticles are entrapped in-between two PTFE layers with given thicknesses. The topographical and morphological characteristics investigated by means of Scanning Electron Microscopy and Atomic Force Microscopy techniques evidenced the very presence of Ag nanoparticles with typical dimension 7 nm. The chemical composition at various depositing steps was evaluated through X-ray Photoelectron Spectroscopy. We show that the presence of the top PTFE layer prevents silver oxidation, while its thickness allows the tailoring of optical properties, as evidenced by spectroellipsometry. The appearance of chemical bonds between silver atoms and PTFE atoms at interfaces is observed, pointing out that despite of pure physical deposition processes, a chemical interaction between the polymeric matrix and metal is promoted by plasma.

## 1. Introduction

The field of optoelectronics is in continuous search for materials with special properties, in order to combine specific optical functionalities with practical considerations such as low weight and easiness of processing. Metal-polymer nanocomposites materials consisting of noble metal nanostructures dispersed in a dielectric polymeric matrix [1] have received considerable attention due to their special physical properties and various applications in optics [2], electronics [3], sensors technology [4,5], as well as life science and biotechnology [6,7].

Inorganic materials are usually characterized by high refractive index (2.0–5.0) [8], but they have disadvantages like higher densities and poor flexibility. Contrary, the organic materials usually have small refractive index (1.3–1.7) [9], but have the advantages of low weight, excellent impact resistance, and good processability compared with inorganic materials [10,11]. Among the most interesting polymers developed lately are those with high refractive index, which find applications in the field of optoelectronics, like optical filters and adhesives, highly reflective [12,13] or antireflective coatings [14], ophthalmic lenses, as well as encapsulants for organic light-emitting diode devices and microlenses for charge coupled devices [10,15]. Thus, the development of organic materials with improved optical properties is a challenging topic.

Polytetrafluorethylene (PTFE) is a widely used polymer for industrial and commercial applications. PTFE possesses high resistance to chemical attack and evidences excellent hydrophobic behavior accompanied by very good anti-sticking properties. With respect to optical and electrical properties, it has good transparency and excellent dielectric properties. PTFE materials have the lowest dielectric constant (2.0–2.1) [16] of any known solid polymers making it very attractive for electronics applications. On the other hand, PTFE exhibit lowest refractive index (1.38) among polymers, which restricts his practical application in the field of optics [17]. Considering this last limitation, the modification of PTFE material by including metal nanoparticles in its structure might be an effective strategy for inducing optical effects which are beneficial for the utilization in optical devices.

Silver nanoparticles have unique thermal, electrical, and optical properties and are widely used in numerous applications [1,5,18]. The most relevant property of the silver nanoparticles with respect to optical applications is the very good efficiency of absorbing and/or scattering visible light due to oscillations of conduction electrons, known as surface plasmon resonance (SPR) effect [5,19]. The combination of plasmonic properties of silver nanoparticles with dielectric properties of PTFE material make silver–PTFE nanocomposites a promising material for optical filters, multilayers, Bragg-reflectors, and sensor applications [20].

Among the methods used for synthesis of metal-polymer nanocomposites, the radiofrequency magnetron sputtering is nowadays a common technique for thin films deposition, starting from solid targets. The physical processes conducting to the removal of atoms in the case of inorganic targets and even larger polymeric fragments in the case of organic targets, followed by deposition to the substrate, bring undoubtable advantages of the RF magnetron sputtering technique over other methods, specifically a high deposition rate, great uniformity on relatively large substrates, as well as the excellent conformality over substrates with various topographies [1,21]. In this work, we report on the modification of PTFE thin films obtained by magnetron sputtering by inclusion of Ag nanoparticles obtained from a second magnetron sputtering source and therefore on the formation of polytetrafluorethylene-silver (Ag-PTFE) nanocomposites. We present the morphological, topographical, and compositional properties of Ag-PTFE nanocomposite materials obtained by this approach, focusing on the influence of the Ag nanoparticles addition on the optical properties and on the effect of a very thin PTFE layer (in the range 5–25 nm) on top of the structure.

## 2. Experimental

### 2.1. Materials and Methods

Polished prime silicon wafers (Topsil Semiconductors sp. z o.o, Warsaw, Poland) of 100 mm diameter and thickness of 440–480 µm, p-doped with Boron and (100) oriented were used as substrates in our experiments. PTFE targets were prepared from PTFE rod (Goodfellow Cambridge Ltd., Huntingdon, United Kingdom) machined according to the technical specifications of the magnetron sputtering source, diameter of 25.4 mm and thickness of 3.18 mm. Silver target (Kurt J. Lesker Company Ltd., East Sussex, United Kingdom) of 99.99% purity with diameter of 25.4 mm and thickness of 3.18 mm was used for silver nanoparticles synthesis. They were cleaned by ultra-sonication in ethanol before mounting on the magnetron head and, before the use in the deposition process, were pre-sputtered in order to clean the surface.

**Figure 1:**
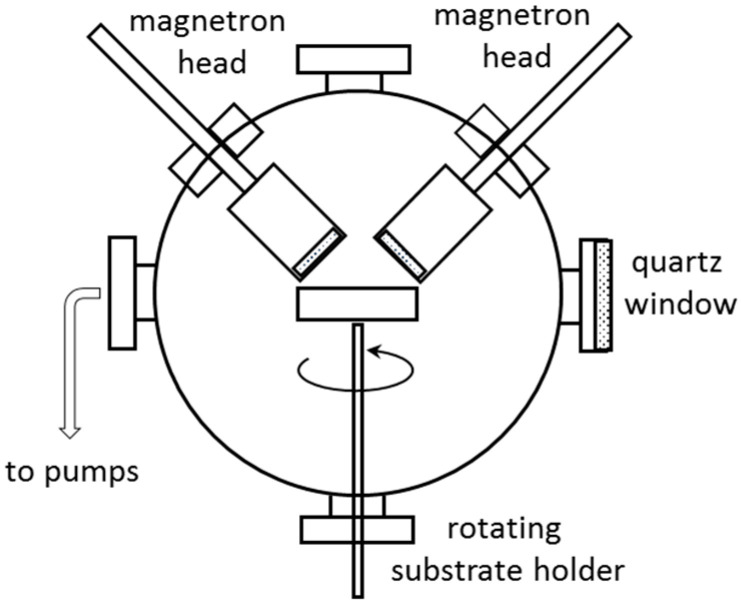
Experimental set-up for polytetrafluorethylene (PTFE)/Ag/PTFE nanocomposites synthesis.

The deposition of PTFE/Ag/PTFE nanocomposites was conducted in a spherical stainless-steel vacuum chamber, equipped with two magnetron sputtering sources (Kurt J. Lesker), positioned at 45° with respect to the substrate holder, as shown in Figure 1. The chamber was evacuated by a system comprising turbomolecular and rotary pumps, down to a base pressure of 1 × 10^−2^ Pa. The pressure in the chamber was monitored by a capacitive gauge (Pfeiffer) and the gas flow rates were controlled by automatic mass flow controllers (Bronkhorst High-Tech B.V., Ruurlo, Nederland). The substrate holder was rotated during the deposition, thus ensuring a good thickness uniformity over the support area. The magnetron sputtering process of both PTFE and Ag were conducted in Ar atmosphere, at a working pressure of 5.5 × 10^−2^ Pa.

**Figure 2:**
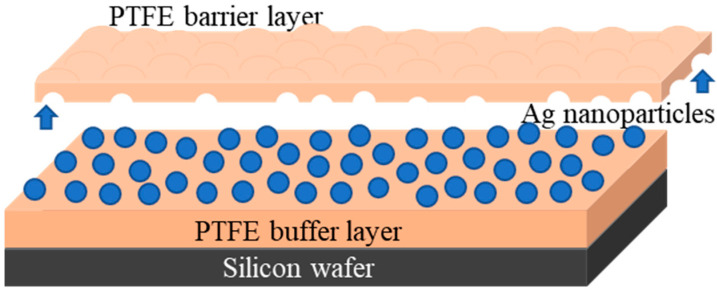
Illustration of the Ag-PTFE nanocomposite materials.

First, a polytetrafluorethylene thin film of 50 nm was deposited onto Si wafers by operating the magnetron source at 80 W RF power. This film, denominated from now on as PTFE buffer layer, was modified by magnetron sputtering deposition of Ag nanoparticles on its surface, upon operating the second magnetron source at 100 W. The exposure time was set for only 3 s, in order to prevent coalescence of Ag islands as a thin film. On top of these, resulted PTFE/Ag nanocomposite were then deposited PTFE layers with thicknesses varied from 5 nm to 25 nm, denominated here as barrier layers since they prevent the Ag oxidation, as we will show later on. The schematic representation of the as prepared PTFE/Ag/PTFE nanocomposites is presented in Figure 2.

### 2.2. Characterization Techniques

The investigation of PTFE/Ag/PTFE surface morphology was performed by using a field emission scanning electron microscope (FE-SEM) MERLIN Compact Base Unit with GEMINI I column from Carl Zeiss, Oberkochen, Germany, using an acceleration voltage of 10 kV. The dimensional distribution and average size of silver nanoparticles diameter were determined by using the ImageJ software (v1.53c version, National Institutes of Health (NIH Image), Washington, DC, USA), designed for scientific multidimensional images processing.

The surface topography of the as-prepared samples was investigated by Atomic Force Microscopy (AFM) (Park Systems Corp, Suwon, Korea) AFM images of 256 × 256 pixels were recorded with a Park Systems microscope, model NX10, operating in non-contact mode utilizing cantilever Hi’res C 14/Cr Au, for areas of 250 × 250 nm^2^ and scan rate of 2 Hz.

Investigations regarding the chemical composition of the resulted PTFE/Ag/PTFE nanocomposites surface were conducted by using the X-ray Photoelectron Spectroscopy (XPS) method. XPS analyses were performed on a K-Alpha Thermo Scientific (ESCALAB™ XI+, East Grinstead, UK) spectrometer equipped with a 180° double focusing hemispherical analyzer. Peak position was calibrated according to the standard C1s peak (284.8 eV). Survey spectra were recorded at a pass energy of 50 eV to determine the elemental composition of the PTFE/Ag/PTFE nanocomposites surface. High-resolution spectra for C1s, F1s, and Ag3d binding energy regions were measured at a pass energy of 20 eV in order to evaluate the elemental bonding states of the as-resulted materials. The spectra acquisition and data processing were performed by using the advanced Avantage data software.

A Woollam Variable Angle Spectroscopic Ellipsometer (WVASE 32, J.A. Woollam Company, Inc, Lincoln, NE, USA) equipped with a HS-190 monochromator with xenon lamp system was involved to measure the amplitude Ψ and the phase difference Δ upon light reflection on the as-obtained surfaces. The refractive index and extinction coefficient dispersion curves of the pure PTFE and PTFE-Ag-PTFE nanocomposites thin films were obtained upon optical modelling of the obtained curves, using either Cauchy model in the case of simple PTFE layers or optical oscillators model for the complex structure of PTFE/Ag/PTFE nanocomposites.

## 3. Results and Discussion

### 3.1. Chemical Composition of the PTFE/Ag/PTFE Nanocomposites Surface (XPS)

**Figure 3:**
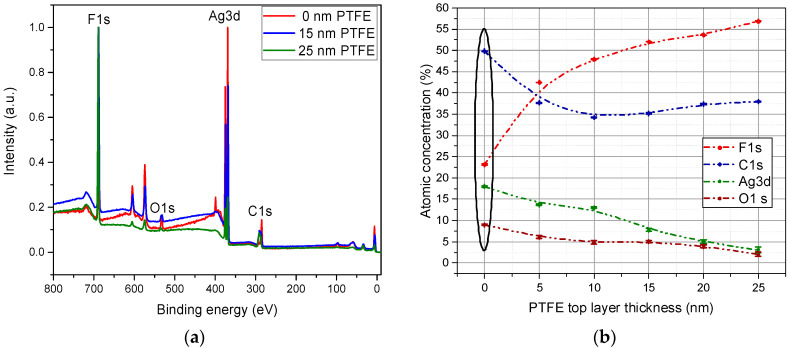
Survey spectra for PTFE/Ag/PTFE nanocomposites as a function of topcoat layer thickness (**a**) and the variation of the atomic concentration of the main elements regarding the thickness of the PTFE topcoat layer (**b**).

X-ray Photoelectron Spectroscopy (XPS) survey spectra revealed on the nanocomposites surface the presence of carbon, fluorine, and silver as the main elements for all investigated experimental parameters (Figure 3a), while oxygen is also present at the surface as an impurity originating either from the surface oxidation after deposition, or by the inclusion in the material during deposition process. The XPS peaks related to Ag 3d decrease with increasing of PTFE barrier layer thickness, while the F1s XPS signal shows an opposite behavior, increasing with PTFE top layer thickness. The dependence of the atomic concentration of elements as a function of the PTFE topcoat layer thickness, as determined from the XPS survey spectra processing are also presented in Figure 3b. The elemental concentration of the Ag-PTFE nanocomposites without the presence of buffer layer, illustrated in the oval in Figure 3b, will be discussed separately, since different mechanisms bring contribution to the overall elemental values in comparison with the materials covered by a PTFE top layer.

As such, for the samples without barrier layer one may notice a higher concentration of carbon and oxygen, which is explained by the processes taking place simultaneously on the Ag surface; namely, on one hand, the oxidation of silver particles due to exposure to atmosphere, and on the other hand surface covering with superficial carbon of contamination, which is even more pronounced in the presence of oxygen [22]. Another reason for this higher concentration of carbon is that the X-ray photoelectron spectroscopy investigations were performed after scanning electron microscopy; this was reported to conduct to surface charging of the sample and thus to increasing the affinity of contamination carbon on the surface [23]. For the materials obtained by applying the barrier layer, the carbon signal presents a monotone behavior regardless the thickness of the PTFE topcoat layer, with an average atomic contribution of 35%.

The fluorine concentration increases in accordance with the PTFE topcoat layer thickness increasing, being 23% for the uncoated silver nanoparticles and reaching around 57% for the PTFE top coated layer of 25 nm thickness. The lower contribution of fluorine atom in the case of the uncoated PTFE/Ag nanocomposites is reasonable in this case as long as a significant part of the PTFE buffer layer surface is covered by the silver nanoparticles, and the XPS signal from underneath Ag NP is most probably diminished. The atomic concentration of silver atom presents a descendant trend starting with a contribution as high as 18% for uncoated PTFE/Ag down to a contribution as low as 3% for the 25 nm PTFE top coat layer, suggesting the successful coating of the silver nanoparticles with PTFE layer. At the same time, the fact that even upon depositing layers much thicker than the escaping depth of photoelectrons (of max 5 nm) suggests that the polymer is preferentially deposited as a matrix in which Ag nanoparticles are embedded. The atomic concentration of oxygen drastically decreases from the uncoated PTFE/Ag nanocomposites from the 9% contribution to 2% in the case of 25 nm PTFE top layer thickness. In the case of uncoated Ag nanoparticles, the contribution of oxygen atom originates from the silver oxidation after air exposure of the as-synthetized silver nanoparticles and just an insignificant contribution comes from the residual oxygen from the deposition process. The deposition of a very thin PTFE layer of 5 nm thickness deposited on top of silver nanoparticles are conducive to a drastic decrease of O1s signal from 6% to 2% for 25 nm PTFE topcoat layer, suggesting a very low oxidation degree of metallic particles in this case. Therefore, we can presume that the PTFE topcoat layer acts as a protective barrier for silver nanoparticles against atmospheric oxygen attack. Additional insight will be provided upon interpretation of the high resolution XPS spectra.

**Figure 4:**
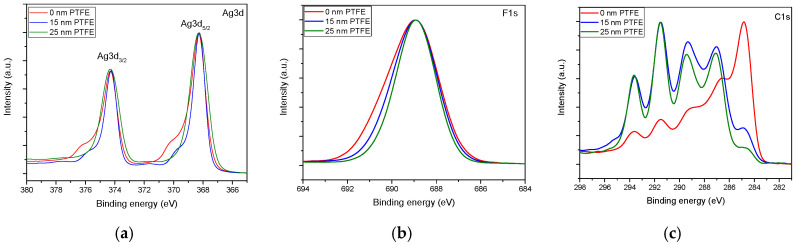
Evolution of the high-resolution spectra for various thickness of the PTFE barrier layer in the binding energy regions associated with (**a**) Ag 3d, (**b**) F 1s, and (**c**) C 1s.

The high-resolution spectra for C1s, Ag 3d, and F1s recorded for the PTFE top layer thickness of 0 nm, 15 nm, and 25 nm elements of interest, which reveal significant variation with respect to the PTFE top coat layer thickness are displayed in Figure 4. For a better comparison of the modifications induced by the barrier layer, all the spectra were normalized.

The Ag 3d spectra present a shoulder towards more negative binding energy in the case of no barrier layer, and this diminishes with the increase of top PTFE layer. At the same time, in the F1s binding energy region, one may notice that the broadest peak is obtained for the material without the top layer, while upon coating the Ag NPs with the PTFE like layer, the peak becomes narrower, suggesting a purer Teflon-like environment. Significant modifications appear as well in the C1s binding energy region, from a contamination-dominated surface towards a typical PTFE-like surface. So, the increasing of PTFE barrier layer thickness is conducive to a pronounced fluorination of the nanocomposites surface and to a drastic decrease of the XPS signal, originating from the photoelectrons emitted by Ag 3d, which suggests the complete covering of silver nanoparticles and thus high protection of these against the attack of atmospheric oxygen.

**Figure 5:**
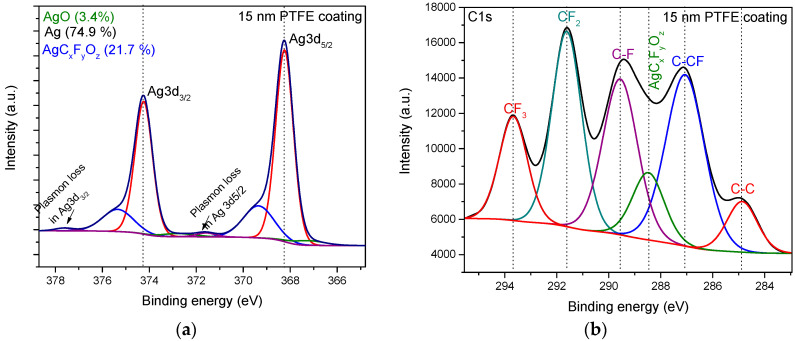
High-resolution spectra of (**a**) Ag 3d and (**b**) C 1s binding energy regions for 15 nm thickness of PTFE topcoat layer and their corresponding deconvolutions presenting the bonding types in the nanocomposite material.

Typical deconvolutions of the C1s and Ag3d_5/2_ high-resolution spectra of the samples covered by the PTFE barrier layer are displayed in Figure 5. The Ag 3d spectrum was fitted with three doublets, Ag 3d_5/2_ and Ag 3d_3/2_, separated by 6 eV, in which the bonds correspond as follows: The most significant contribution of 74.85% corresponds to metallic silver (368.2 eV), while a very low contribution of 3.38% is assigned to AgO bonds (367.2 eV) [24,25], suggesting the insignificant oxidation process in the nanocomposites synthesis process. The third peak, positioned at 369.4 eV, with a significant contribution of 21.77% in the case of 15 nm barrier layer, was assigned to AgC_x_F_y_O_z_ (x > 2, y < 3) type bonds considering a more pronounced shift with respect to the AgC_2_F_3_O_2_ bond reported in reference [25] at the binding energy of 368.8 eV. Indeed, in our case, one would expect a higher carbon content with respect to the AgC_2_F_3_O_2_, leading to a corresponding energy shift. Finally, a very small doublet peak at high energies (371.8 eV) evidence the plasmon loss contribution, proving the nanostructured character of Ag in the dielectric matrix of PTFE.

The deconvolution of C1s spectrum for silver containing-PTFE-like material was performed using six components which correspond as follows: A very low contribution of 6.3% is devoted to C-C bond (284.8 eV); a contribution of 27.1% is assigned to C–CF bond (287.0 eV); a contribution of 22.1% for CF bond (289.5 eV). An important contribution of 23.9% is assigned to CF_2_ bond (291.5 eV), while a lower contribution of 11.9% is devoted to CF_3_ bond (293.6 eV) [1,21,26,27,28]. Besides the five peaks typically obtained in the PTFE-like thin films obtained by magnetron sputtering deposition [29,30], an additional peak with a contribution of 8.7% was considered during the fitting procedure, and this is associated with AgC_x_F_y_O_z_-type bond (288.4 eV) [25].

**Figure 6:**
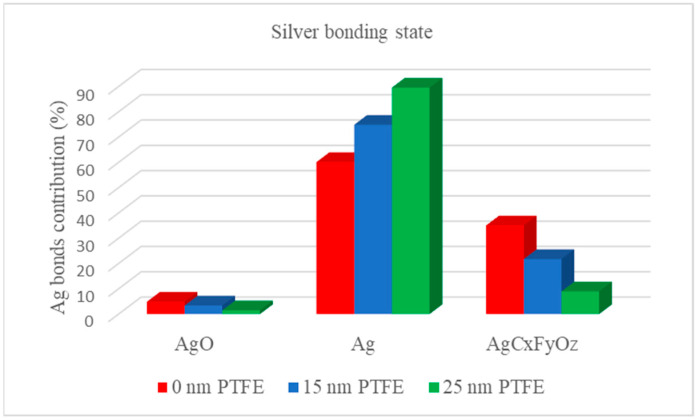
The evolution of Ag- related bonds as function of the PTFE barrier layer.

The processing of high-resolution carbon spectrum reveals the complex structure of the as-synthetized nanocomposite materials, with a significant contribution of the carbon-related bonds specific to PTFE-like material and just a low contribution of a complex carbon-silver bond, resulted from the interaction of Ag material with the PTFE layer, obtained by the combined magnetron sputtering deposition. The evolution of the Ag-related components as resulted upon deconvolution of the XPS spectra for the Ag 3d region for various thicknesses of the PTFE barrier layer aspect is illustrated by Figure 6. The results evidence the diminishing of the AgC_x_F_y_O_z_-type bond when the thickness of the top barrier layer increases, revealing that this bond actually comes from the interface between the initial PTFE buffer layer and the Ag nanoparticles. It points out toward an interaction of the arriving Ag species onto the freshly prepared PTFE layer, which leads to chemical bonding between them. On the other hand, the metallic silver content increasing with the PTFE barrier layer thickness. This effect proves that the PTFE top coating acts as protection barrier allowing the use of these materials as optical filters by photooxidation prevention insured by the PTFE layer.

### 3.2. Morphological Characteristics

**Figure 7:**
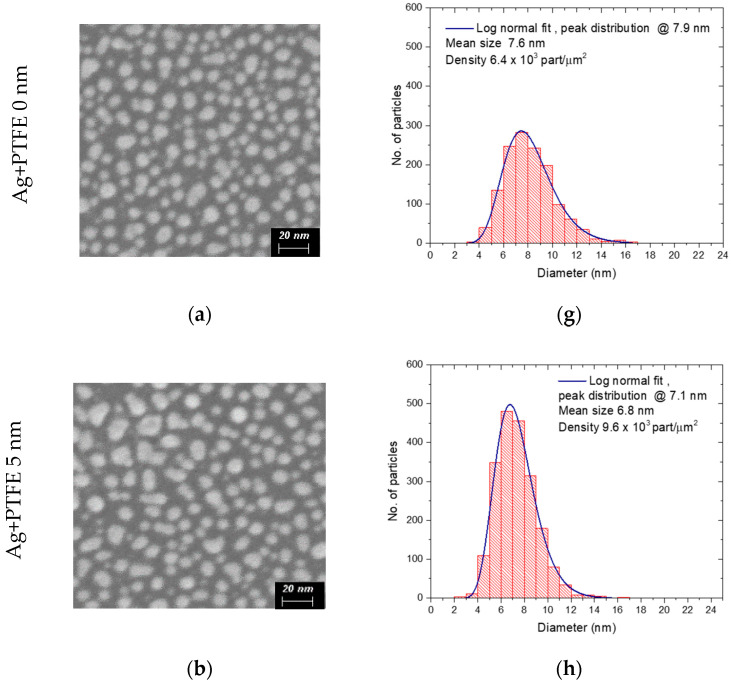

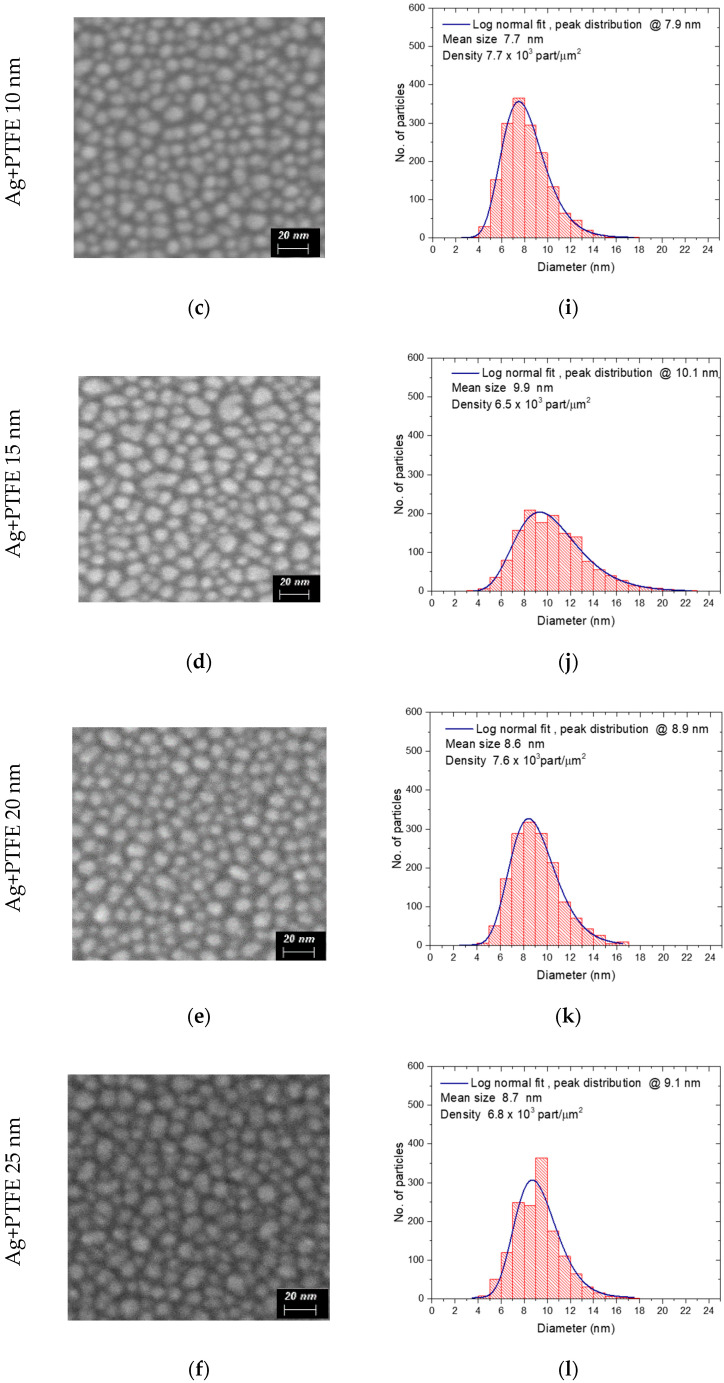
(**a**–**f**) HR-SEM images of the Ag-PTFE nanocomposites with various thicknesses of the top layer; (**g**–**l**) size distribution of the nanoparticles (NP) observed in HR-SEM images.

Scanning electron microscopy (SEM) images of the PTFE/Ag/PTFE nanocomposites surface obtained for various thicknesses of PTFE topcoat layer are shown in Figure 7. The size distribution, particles density, and average diameter of the as-prepared PTFE structures with silver nanoparticles inclusions as a function of PTFE topcoat layer thickness are also presented. The dimension of silver nanoparticles was measured from SEM images, by using ImageJ program, for the analyzed area of 0.212 µm^2^.

The SEM images reveal the presence of dense features, associated with the lighter signal, with similar appearance regardless of the thickness of the PTFE barrier layer. We associate them with Ag nanoparticles, hosted in the Teflon-like polymeric matrix formed by the PTFE layers deposited by magnetron sputtering. For the sample consisting only of PTFE buffer layer modified by silver nanoparticles (Figure 3a) round to oval-like shapes nanoparticles are present on the surface showing a log normal distribution and dimensions varying from below 5 nm up to 17 nm. In most of the cases, the larger nanostructures present more irregular shapes evidencing a formation mechanism, based on a coalescence of adjacent silver nanoparticles. For the 5 nm PTFE topcoat layer, the silver nanoparticles have more regular shape, a narrow size distribution with particle mean diameter of 6.8 nm, as well as an increased density on the surface. This phenomenon can be explained by the deposition process of the barrier layer immediately upon Ag sputtering, which somehow stabilize the Ag nanoisland in their position and prevent their further nucleation, as might be the case for the uncoated Ag-PTFE structure [31]. As the PTFE top layer thickness is increased, the particles’ density slightly decreases, but their size becomes larger and the distance between them get shorter. The increase of the average diameter indicates the covering of the Ag nanoparticles with a thin PTFE layer as well as the fact that the PTFE deposition takes place inside the spaces between them, as previously shown in the XPS investigations.

**Figure 8:**
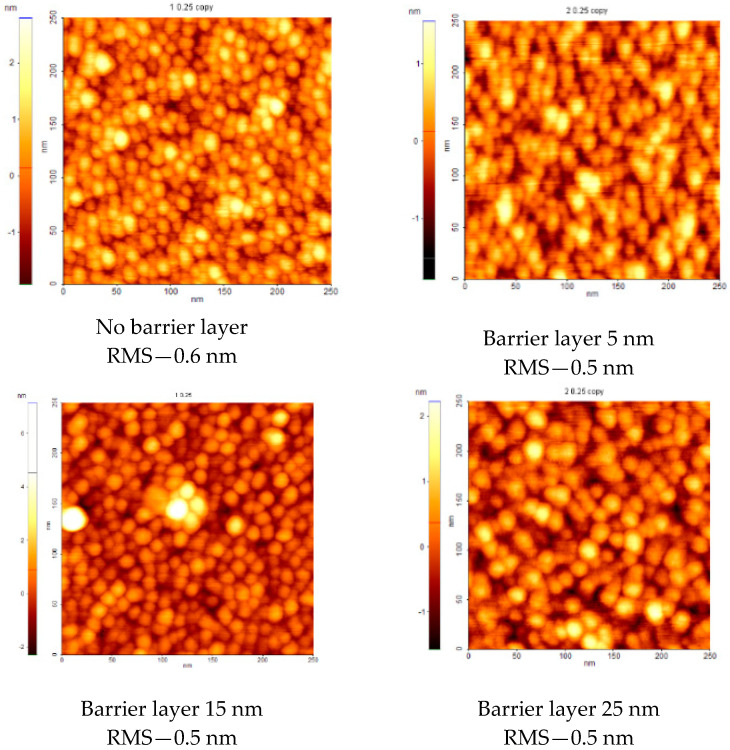
AFM images of the PTFE/Ag/PTFE nanocomposites.

In order to check the surface topography, AFM measurements have been performed on 250 × 250 nm areas and are presented in Figure 8 for PTFE/Ag/PTFE nanocomposites materials with PTFE buffer layers of 0, 5, 15, and 25 nm.

The AFM technique indicates a similar information as shown by SEM, namely the presence of rounded nanoparticles on the surface. Although their apparent diameter increases with PTFE buffer layer thickness, the surface is extremely smooth presenting RMS roughness values below 1 nm in all investigated samples. A very slight decrease from 0.6 nm in the case of uncoated Ag nanoparticles to 0.5 nm in all the other cases indicate a smoothening effect given by the buffer layer deposition, which enhance the effective refractive index and therefore favor the light reflection properties.

### 3.3. Optical Characterization of the PTFE/Ag/PTFE Nanocomposites

The spectroscopic ellipsometry data (ψ and Δ) have been experimentally measured at an angle of incidence of 60°–70° of the light beams in the spectral range from UV-vis region to near-IR region (250 to 1700 nm). Four materials layers were considered for simulating the ellipsometric data: The silicon substrate, the native silicon oxide (3 nm thickness), the Ag-PTFE nanocomposite layer, and a layer counting for the sample roughness. The Cauchy–Urbach model was applied for calculating the optical constants in the case of pure PTFE layer, while in the case of materials consisting of silver, the general oscillator’s model was applied. The top rough layer was considered to be composed by a mixture of voids with Ag-PTFE in equal proportion (50:50) and was calculated using Bruggeman effective medium approximation (BEMA) [32].

**Figure 9:**
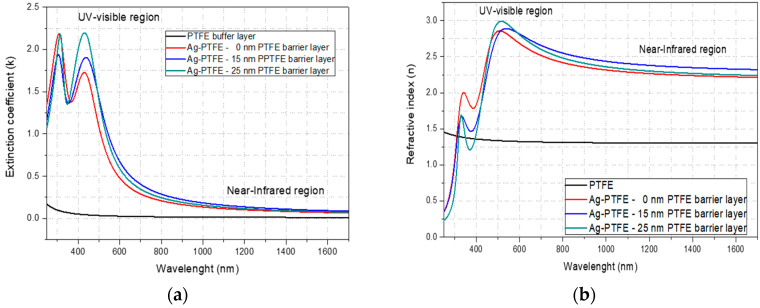
Dependency of the extinction coefficient (**a**) and refractive index (**b**) upon the wavelength for pure PTFE material and Ag-PTFE nanocomposites with various thickness of PTFE top coat layer.

The optical constants of the Ag-PTFE nanocomposites with various thicknesses of the PTFE topcoat layer are comparatively presented in Figure 9.

The magnetron sputtered PTFE-like material does not present any absorption along the entire investigated wavelength domain, from the UV-Vis region to near IR region. Instead, a clear absorption peak around upon 430 nm, associated with the surface plasmon resonance effect due to the presence of Ag nanoparticles in the PTFE matrix, observed in the case of the Ag-PTFE nanocomposites [33]. The absorption coefficient increases with the PTFE top layer thickness, from 1.7 in the case of the uncoated silver nanoparticles to 2.2 in the case of 25 nm PTFE topcoat layer thickness. This extinction increasing could be explained by the presence of the PTFE top coating layer. Considering that the silver nanoparticles present similar dimensions and the density of these on the surface is in the same order of magnitude, the PTFE top coat layer is most probably responsible for negligible scattering of the incident light. The enhancement in the SPR peaks can be related to the dielectric constant of the host material. The position and width of the SPR is by dielectric functions of both the Ag and the PTFE as surrounding media [34] The thickness dependence of dielectric constant of PTFE can explain the difference of SPR width. According to Hsu et al., the values of dielectric constant for PTFE is lower when the thickness is lower [35] and in this case, the width of SPR is higher for the sample with high thickness of PTFE layer. However, additional theories must be involved in order to efficiently compute the scattering and absorption coefficients of our spherical silver nanoparticles [36].

Our obtained materials present strong absorption and/or scattering behavior upon interaction between electromagnetic radiation and the conduction electrons known as surface plasmon resonance effect (SPR), making them suitable for using as optical biosensors [37,38,39]. The refractive index calculated for magnetron sputtered PTFE material present value below 1.5 and is comparable with the refractive index value of the initial PTFE target (*n* = 1.38) according to the technical specifications of the supplier [40]. In the case of the metallic nano-silver inclusion in the PTFE matrix, an important increasing of the refractive index is obtained, up to 2.7 at 632 nm, and ~2.4 for 700–1700 nm range (near-IR domain) rendering the Ag-PTFE nanocomposite highly suitable for optical applications from visible region to near-infrared spectrum. The as-synthetized Ag-PTFE nanocomposites present significant refractive properties, reaching higher refractive indices in respect to other work relevant for this field [11,14]. The obtained high refractive index in near-infrared spectrum can provide the possibility of using of these materials as optical media with controllable refractive index for a broad wavelengths range, including visible region [41]. Moreover, the spectroellipsometry fit reveals that such optical constants could not be obtained by simply stacking continuous layers of PTFE and Ag, suggesting the importance of nanocomposites with embedded Ag NPs for optical applications.

## 4. Conclusions

In this work, we successfully synthesized Ag-PTFE nanocomposites with various thicknesses of the top PTFE layer, in the range 5–25 nm, by means of successive magnetron sputtering of Ag and PTFE targets. Almost spherical silver nanoparticles are present in the nanocomposites, with typical dimensions around 7 nm, while the overall roughness RMS values remain as low as 0.5 nm in all investigated cases. The XPS results evidence the formation at interface of AgC_x_F_y_O_z_ bonds upon the arrival of Ag atoms onto the freshly deposited PTFE buffer layer. The top PTFE layer ensures the stabilization of the Ag spherical nanoparticles in the structure. At the same time, it ensures an effective barrier against silver oxidation, as evidenced by the large amount of metallic silver in the structure. The obtained surface plasmon resonance peak around 430 nm is further enhanced with the PTFE layer thickness. The overall optical properties allow us to conclude that the produced nanocomposites materials with surface plasmonic resonance are suitable for biosensors design, as well as antireflective coatings for a wide variety of technological applications, from optical filters to windows, and other optoelectronic devices in which the reflections limit the performance of the devices.
